# Advances in Fabric-Based Pneumatic Soft Actuators for Flexible Robotics: Design and Applications

**DOI:** 10.3390/s25123665

**Published:** 2025-06-11

**Authors:** Yao Chai, Yutong Qin, Ziyi Xu, Xianhong Zheng, Hao Jia

**Affiliations:** 1 Key Laboratory of Eco-Textiles, Ministry of Education, Jiangnan University, Wuxi 214122, China; 2 School of Textile and Garment, Anhui Polytechnic University, Wuhu 241000, China

**Keywords:** pneumatic soft actuators, fabric, flexible robotics, reversible Zn metal electrodeposition

## Abstract

As a groundbreaking innovation in the field of soft robotics, fabric-based pneumatic soft actuators exhibit substantial advantages over traditional rigid mechanical systems in terms of adaptability, safety, and multifunctionality. This paper presents a thorough review of the design principles, classifications, and application advancements of these actuators. By leveraging the intrinsic flexibility and programmability of fabric materials, these actuators achieve complex and precise motion control through the modulation of internal air pressure. This review investigates the state-of-the-art research progress in overcoming critical challenges, such as enhancing multidirectional expansion capabilities, optimizing the trade-off between flexibility and driving force, and improving control accuracy and response speed. Furthermore, the integration of fabric-based actuators with flexible sensors is highlighted as a highly promising research direction, offering the potential to enhance device intelligence via real-time feedback and adaptive control functionalities. In conclusion, with ongoing advancements in material science, structural design, and control strategies, fabric-based pneumatic soft actuators are expected to unlock broader application potentials in domains such as healthcare, wearable technology, and human–-computer interaction.

## 1. Introduction

In the rapidly evolving landscape of modern engineering and robotics, soft actuators have emerged as a groundbreaking innovation. They serve as advanced driving devices in the field of flexible robotics and beyond, capable of inducing material deformation, motion, and corresponding mechanical responses through a variety of external stimuli such as electrical [1], magnetic, optical changes [2], or internal flow fields [3]. Among these, pneumatic soft actuators hold a particularly significant place due to their unique advantages. They are especially important when designing robots that need to be highly flexible or made from fabric materials. Pneumatic soft actuators can provide gentle yet powerful actuation forces, making them ideal for applications where delicate interactions are required, such as in medical or caregiving robots that need to handle fragile objects or human tissues with care. Moreover, their lightweight and compliant nature allows for seamless integration into fabric-based robotic systems, enhancing the overall adaptability and performance of the robots in complex environments. The design and functional implementation of these actuators provide an innovative alternative to traditional mechanical drive systems dominated by rigid structures [4]. Compared with classical motor or hydraulic drive systems, soft actuators, including pneumatic ones, exhibit significant advantages in terms of weight, precision, and environmental adaptability [5], particularly in confined spaces [6] or environments with specific requirements [7]. The inherent flexibility of soft materials not only introduces novel design concepts for engineering applications but also stimulates profound reflection and innovation regarding the design and operational principles of existing mechanical systems [8].

In terms of diversity and innovative design, the range of soft actuators continues to expand, encompassing electroactive polymers [9], photo-actuated actuators [10], and ion-driven actuators [11], each optimized for specific application needs. These designs not only enhance actuator performance but also significantly broaden their applicability across various domains [12]. Especially when integrated with sensors [13], the potential of soft actuators in robotics, adaptive structural design [14], and sensing detection [15] has been further demonstrated [16]. This integrated approach enables soft actuators to perform predetermined movements while simultaneously monitoring and adapting to their operating environment in real time, thereby playing a pivotal role in automation, medical devices [17], environmental monitoring, and wearable technology [18].

With the continuous advancement of material science [19], micro- and nano-fabrication technologies, and intelligent control algorithms [20], the performance and functionality of soft actuators continue to improve and expand. Future research will focus on the development of novel stimulus-responsive materials, innovations in actuation mechanisms, and enhanced compatibility with biological tissues to enable more complex and precise motor control. Additionally, the multidisciplinary nature of soft actuators suggests that their application prospects in the simulation of biological systems [21], human–computer interaction [22], and complex operational environments will be even broader [23], potentially revolutionizing the future development of soft robotics technology.

## 2. Overview of Soft Actuators

### 2.1. Principles and Classification of Soft Actuators

Soft actuators, as core components in soft robotics technology, can be classified in detail based on the external stimuli they respond to. These stimuli include, but are not limited to, changes in liquid pressure, temperature gradients, electric fields, and magnetic fields. Each stimulus source endows soft actuators with unique driving mechanisms and application potential.

(1) Liquid-driven Soft Actuators

Liquid-driven soft actuators typically rely on liquid flow and pressure changes to achieve movement. The core of such actuators is a flexible yet elastic cavity filled with liquid (usually oil or water). When liquid is injected or withdrawn from the cavity, the cavity expands or contracts accordingly, driving the movement of the entire device. The advantage of this type of actuator lies in its ability to generate significant force and high energy density, making it suitable for applications requiring high force output, such as flexible robotic arms or bionic devices [24].

(2) Temperature-driven Soft Actuators

Temperature-driven soft actuators are innovative devices that utilize temperature changes as a power source. These actuators convert thermal energy into mechanical motion energy through the thermal expansion effect caused by temperature differences, driving the soft actuator to perform specific tasks [25]. By taking advantage of the thermal expansion and contraction properties of materials, temperature changes can cause changes in the shape, length, or volume of the soft actuator, thereby achieving mechanical motion. Temperature-driven soft actuators have the advantages of simple structure, fast response speed, and no need for external power sources. They show great potential in micro-mechanical systems, biomedical devices, and soft robotics. As an environmentally friendly and pollution-free driving technology, temperature-driven soft actuators may become important driving devices in various application fields in the future, bringing more innovation and application possibilities.

(3) Magnetic-driven Soft Actuators

Magnetic-driven soft actuators achieve movement through the interaction between magnetic fields and magnetic materials [26]. These actuators typically contain magnetic particles (such as ferrite particles), and when the external magnetic field changes, these particles rearrange, causing the material to deform. The advantage of magnetic-driven actuators is their simple control and fast response speed, allowing for precise motion control. Additionally, since the magnetic field can be applied remotely without contact, these actuators are particularly suitable for environments where direct contact is not possible.

(4) Electric-field-driven Soft Actuators

Electric-field-driven soft actuators (also known as electroactive polymer actuators) change the shape of polymers by applying an electric field [27]. These actuators are typically composed of dielectric materials and conductive electrodes. When an electric field is applied, the attraction or repulsion of charges between dielectric materials causes the material to expand or contract. The advantages of electric-field-driven actuators include high energy efficiency and low driving voltage, making them highly suitable for applications requiring fine control and rapid response.

In addition, multi-response actuators represent a significant advancement in the field of soft actuators [28,29]. These actuators are capable of responding to diverse stimuli, including electric and magnetic fields, as well as electrical and optical signals. Compared with single-stimulus actuators, their adaptability and functionality are markedly enhanced [30,31]. They can integrate multiple sensing and actuating mechanisms into a unified system, enabling complex and precise control. As such, they find extensive application in robotics, wearable devices, and biomedical engineering. This integration offers numerous advantages. For example, the combination of electrical and magnetic stimulation enables high-speed response and robust force output [32]. Optical response introduces non-contact remote control capabilities, thereby expanding the operational range of the actuator while enhancing safety and user-friendliness [33]. Nevertheless, the development of multi-response actuators presents several challenges. The design and fabrication processes must comprehensively address material selection, structural optimization, and the integration of multiple mechanisms. Additionally, the control system must be sophisticated enough to coordinate multi-signal inputs and responses effectively [34]. Despite these challenges, the potential benefits of multi-response actuators are substantial. They are expected to drive advancements in intelligent multi-functional soft robotic systems, environmentally adaptive wearable devices, and efficient personalized biomedical devices. Future research should focus on overcoming existing limitations, exploring novel materials and design strategies, and fully realizing their potential.

### 2.2. Principles of Pneumatic Soft Actuators

Pneumatic soft actuators are similar to liquid-driven actuators and are one type of fluid-driven actuators. These actuators adjust the volume of the cavity by controlling the injection and release of gas, thereby achieving structural deformation or movement. Pneumatic soft actuators typically contain one or more inflatable chambers. When gas (usually air or inert gas) is pumped into the flexible chambers of the actuator, the pressure inside the chambers increases, causing the chambers to expand. The expansion deformation is then converted into stretching, bending, twisting, and other motion forms through geometric asymmetry or material anisotropy. Conversely, when the gas is released, the chambers contract, and the actuator returns to its initial position or generates motion in the opposite direction.

Therefore, each type of soft actuator has unique advantages and challenges, as illustrated in Table 1. Liquid-driven actuators can produce significant force and high energy density, making them suitable for applications requiring substantial mechanical output, but they require complex fluid systems and have slower response times. Temperature-driven actuators are simple, fast, and can operate without external power, making them ideal for autonomous or environment-powered systems, yet their performance is highly dependent on ambient temperature and material thermal conductivity, limiting their precision and reliability in variable environments. Magnetic actuators offer precise control and fast response with remote operation capabilities, but they need a magnetic field generator and may interfere with other devices. Electric-field-driven actuators, such as electroactive polymers, provide high energy efficiency, low driving voltage, and fine control with rapid response, but they are sensitive to environmental factors like humidity and temperature, and require complex electrical systems. The choice of soft actuator depends on the application requirements, including force output, response speed, environmental conditions, and available power sources. Understanding the pros and cons of each type is crucial for optimizing their use in different fields. The main advantages of pneumatic soft actuators lie in their flexibility and safety. Compared with traditional motor or hydraulic drive systems, they are lighter, respond faster, and can work in narrow or hard-to-reach environments. Additionally, due to the compressibility of gas, pneumatic systems can provide more precise control while reducing energy consumption and enhancing system safety.

### 2.3. Challenges Faced by Pneumatic Soft Actuators

With the continuous advancement of technology, pneumatic soft actuators are expected to play a key role in more fields, promoting the development of soft robotics and automation systems. Despite their many advantages, pneumatic soft actuators also face numerous challenges in design and application, such as control accuracy, response speed, and durability. Future research may focus on developing new materials, improving pneumatic network design, and integrating intelligent sensors to enhance the performance and application range of pneumatic soft actuators. Currently, pneumatic soft actuators mainly face the following three challenges:

(1) Challenges of Multi-directional Expansion and Large Deformation in “Balloon-like” Structures

Pneumatic soft actuators rely on the expansion and contraction of flexible materials under internal pressure to generate motion, but precisely controlling this expansion and contraction is very difficult. The nonlinear characteristics of flexible materials increase the complexity of predicting and regulating their deformation at different pressures. To achieve directional expansion in a specified direction, the internal structure design must be precise, which involves complex pneumatic network design and fine manufacturing techniques to ensure uniform pressure distribution and precise control. Moreover, materials may experience fatigue and damage when undergoing large deformations, thereby affecting the durability and reliability of the actuator. Therefore, researchers need to develop new materials and design strategies to enhance the stability and durability of the actuator while maintaining its flexibility and adaptability.

(2) The Contradiction Between Inherent Flexibility and Driving Force

Although flexible materials can provide soft interaction and good environmental adaptability, they often lack sufficient rigidity to transmit high driving forces. This contradiction is particularly evident in applications where the actuator needs to perform heavy-load operations or precise control. For example, in the grasping and manipulation tasks of soft robots, the actuator needs to provide sufficient force to hold objects firmly without damaging sensitive surfaces.

(3) Insufficient Control Accuracy and Response Speed

Pneumatic soft actuators encounter challenges in achieving high precision and fast response, mainly due to the compressibility of gas and the hysteresis of flexible materials, which typically result in lower response speed and control accuracy compared to electric or hydraulic systems. Gas flow delay and uneven pressure distribution may cause unsmooth and inaccurate motion, especially in applications requiring rapid and precise control, such as medical surgeries or precision assembly. Additionally, the nonlinear behavior and unpredictable deformation of flexible materials further increase the complexity of control.

## 3. Overview of Pneumatic Soft Actuators

Since 2010, pneumatic soft actuators have become the focus of many researchers, generating widespread research interest. Researchers worldwide have successfully developed and demonstrated various pneumatic soft actuators with innovative structures, significantly promoting the development of soft robotics technology. The design diversity of these actuators is reflected in their structural forms, with some typical designs including fiber-reinforced structures, elastic chamber structures, corrugated structures, folded or wrinkled structures, etc. These different structural designs not only expand the application range of pneumatic soft actuators but also exhibit unique motion characteristics and functions, providing customized solutions for specific application scenarios. These research achievements not only demonstrate the deep integration of multiple disciplines such as material science, mechanical engineering, electrical engineering, and computer science, but also provide new ideas and methods for the design and manufacturing of soft robots.

(1) Fiber-reinforced Pneumatic Soft Actuators

**Figure 1:**
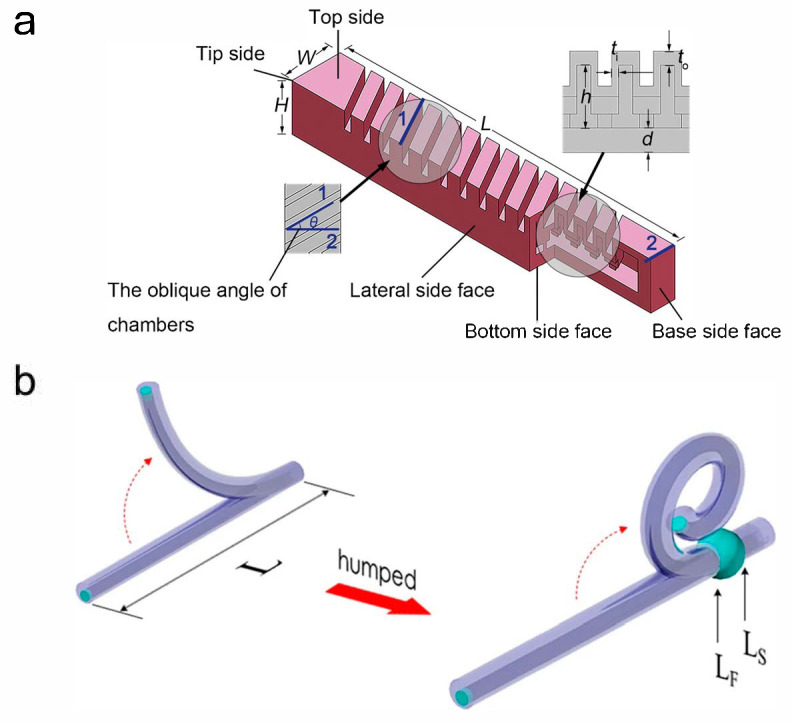
(**a**) The structure of a pneu-net actuator with oblique chambers (Edge 1 represents the inclined side of the actuator cavity, edge 2 represents the width side of the actuator, and edges 1 and 2 form the cavity angle) [38]. (**b**) A schematic diagram of PDMS microtube tentacle actuator. Unlike the plain microtube (left), the one shape-engineered with a hump (right) can produce a tentacle-like spiraling motion. Fabrication steps can be found in [39].

Fiber-reinforced pneumatic soft actuators mainly present anisotropic mechanical properties by covering or embedding fibers, fabrics or other similar structures on or in the cavity structure. They have a relatively simple structure, are easy to manufacture and can provide a large end force. In the 1950s, researchers have successively proposed the concept of fiber-reinforced pneumatic soft actuators, which are also known as McKibben artificial muscles or pneumatic artificial muscles (PAMs) [35]. On this basis, researchers at home and abroad have conducted in-depth studies on the theoretical modeling, deformation modes and practical applications of fiber-reinforced pneumatic soft actuators. Kanno et al. [36] conducted multiple studies on the integrated sensing technology of traditional McKibben artificial muscles. By embedding dielectric elastomer sensors (DESs) into PAMs, PAM-DES sensor actuators were prepared. Tests on the driving and sensing performance of this PAM-DES sensor actuator revealed that the presence of the sensor had almost no impact on the actuator’s performance, with low hysteresis (drift error of 1.6% at 0% strain) and high repeatability (over 1000 cycles), providing a model reference for the preparation of similar sensor actuators. Abe et al. [37] proposed a new type of active weaving called “18 Weave”, which is more flexible, lighter and can achieve soft power support compared to traditional McKibben artificial muscles. The outer diameter of “18 Weave” is approximately 2 mm. Tests showed that the contraction rate of a single thin McKibben muscle was 22.5%, while that of “18 Weave” was reduced to 26.5%, with a displacement expansion of 19.4%, significantly improving the support effect. Additionally, researchers used ethylene propylene diene monomer instead of ordinary rubber tube materials, significantly enhancing durability and presenting good application prospects in practical fields. Wang et al. [38] A novel structure of pneu-net actuators is proposed, which can achieve coupled bending and twisting motions in three-dimensional space by altering the chamber angle, as shown in Figure 1a. The chamber angle, denoted as α, is defined and its influence on the actuator’s deformation and motion is investigated through finite element analysis (FEA) and experimental validation. During the experiments, all other geometric parameters of the actuators are kept constant while only the chamber angle is varied. Paek et at. [39] have developed a novel direct-peeling technique to fabricate long, thin, and highly deformable microtubes, and established a semi-analytical model for shape engineering. The optimized micro-tentacles can achieve two full turns of spiraling with a final radius as small as 185 μm, enabling them to wrap around and grasp microscale objects (1b). This capability holds significant potential for applications in biomedicine, particularly in cellular manipulation.

On the other hand, under high-pressure gas drive conditions, traditional McKibben artificial muscles are restricted by the mesh fibers, causing radial expansion deformation and generating linear or curved motion in the length direction. To address this, researchers have proposed new improved structures. For instance, Jamil et al. [40] designed a hybrid optical fiber sensing soft pneumatic gripper. Previous studies have shown that rigid optical fibers are suitable for long-distance signal transmission, while flexible optical fibers exhibit better sensing response capabilities in lateral deformation and are often used in extreme environments. Therefore, by alternately using rigid and flexible optical fibers, contact force at specific points can be sensed, and the contact force during deformation can be controlled using a simple PID controller. Additionally, it can be used to locate optical fibers and prevent out-of-plane deformation of the polymer PneuNet, thereby maintaining the high actuation performance of the actuator and showing good application prospects in extreme environments. Yi et al. [41] proposed a new type of pneumatic soft linear actuator, the fiber-reinforced origami robot actuator (FORA). Experiments showed that when actuated by compressed air, FORA produced linear axial contraction motion. At an input pressure of 100 kPa, using the original inner cavity, FORA could achieve 50% of the maximum contraction. Compared with existing McKibben-type actuators, it provided almost twice the range of motion, significantly improved force distribution, and significantly reduced the driving pressure. Na et al. developed a pneumatically actuated soft actuator capable of axial extension, bending and twisting [42], and fabricated a multi-degree-of-freedom flexible manipulator to demonstrate the potential applications of the pneumatically actuated soft actuator.

In addition, the motion of soft actuators can also be achieved by embedding reinforcing structures such as fibers into the elastomer. During the manufacturing process, a group or multiple groups of fibers are wound around the outer surface of the soft cavity, and the fibers and the actuator are combined into a whole through a flexible matrix such as liquid silicone rubber. By designing the winding method of the fibers in the pneumatic soft actuator, the actuator can generate motions such as stretching, rotation, bending, and spiraling. Polygerinos et al. [43] modeled the flexible fiber-reinforced bending actuator in 2015 and developed an accurate and experimentally verified quasi-static computational (FEM) and analytical model for a specific type of flexible actuator—the flexible fiber-reinforced bending actuator. In the same year, the researchers wound the fibers in a spiral manner around the outer surface of the soft cavity to achieve the expansion, stretching, and twisting motions of the pneumatic soft actuator. Changing the spiral angle of the fibers can increase the motion range of the pneumatic soft actuator. Kadir et al. [44] improved the stroke characteristics of the McKibben actuator by using a woven actuator with a nested structure. The common McKibben actuator only has 20% of the total length of the actuator stroke limit [45], which is limited in practical applications. To address this, people have changed the structure of the actuator by weaving and twisting or added additional structures (such as nested or curved structures) to the actuator to increase its stroke. A combined telescopic nested structure and woven actuator was used to increase the contraction ratio of the actuator. During the testing process, the performance of the nested woven actuator (NBA) was compared with that of the single actuator (SA), the woven actuator (BA), and the nested actuator (NA). The results showed that at 350 kPa, the contraction rate of NBA was the highest, reaching 45.5%, followed by NA (39.38%), BA (29.57%), and SA (23.41%). Compared with SA, it can achieve a high stroke with only a 20–30% loss in contraction force, and has good application prospects in practical applications. Due to the strong pressure-bearing capacity of the fiber-reinforced pneumatic soft actuator, it is widely used in bionic robots [46], soft manipulators [47], and wearable devices [48], etc.

(2) Elastic Chamber Type Pneumatic Soft Actuator

**Figure 2:**
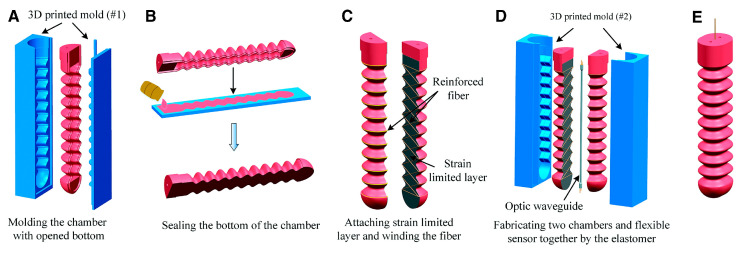
Typical fabrication steps of the soft actuator, structure of the optical waveguide and freely continue bending deformation of the soft actuator. (**A**) Fabricating the open chamber with a sinusoidal shape. (**B**) Closing the open side of the chamber with an elastomer sheet. (**C**) Attaching the strain limited layer and winding the reinforced fiber. (**D**) Encapsulating the two fiber-reinforced chambers and flexible sensor together. (**E**) The final soft actuator with bidirectional bending ability [49].

The motion of the elastic chamber type pneumatic soft actuator mainly utilizes the non-uniform/uniform distribution of materials (or elastic modulus) in space. The elastic chamber type pneumatic actuator has been continuously developed in recent years. By dividing the space inside the chamber, such as bidirectional symmetry along the central axis or trisection, the actuator can undergo different changes when inflated at different parts [49] (Figure 2). Some researchers have added rib structures to the chamber to prevent radial expansion. The straight-through cylindrical elastic chamber is the simplest structure. Zhong et al. [50] designed a bidirectional symmetrical pneumatic soft actuator. The researchers used the lost-wax casting process instead of the commonly used soft lithography technique to manufacture the fixture, which can achieve any shape of internal channel by eliminating the need for lamination. The wrinkled channel design consists of uniformly distributed ribs, shown in cyan, and the embedded hollow parts are shown in yellow. This design is beneficial for grasping because it has high curvature, minimal radial expansion, and remains compliant during the driving process. This design is beneficial for grasping, with high curvature, minimal radial expansion, and remaining compliant during the driving process. Jones et al. [51] utilized interfacial flow in elastomers to gradually solidify them, thereby robustly fabricating monolithic pneumatic actuators whose shapes can be easily customized to suit applications ranging from artificial muscles to various grippers. The flexibility, robustness, and predictability of this method offer promising prospects for assembling complex actuators in terms of geometry, materials, and nonlinearity.

With the continuous in-depth research on elastic chamber-type actuators, more ingenious cavity structures have emerged. Li et al. [52] proposed a bamboo-joint-like soft actuator. In terms of structural design, each “bamboo tube” restricts the radial/circumferential expansion deformation of the actuator; the “spinal cord” restricts the axial stretching deformation of the actuator; and the bamboo nodes increase the bending stiffness of the actuator and enhance the bending effect. Relevant verification tests on this bamboo-joint-like soft actuator revealed that within the range of *p* ≤ 50 kPa, the relative error between the theoretical calculation value and the experimental measurement of the bending central angle of the soft actuator did not exceed 10%, indicating the structural rationality of this bamboo-joint-like soft actuator. Inspired by octopus tentacles, Xie et al. [53] attempted to design a unique conical soft actuator. Compared with traditional cylindrical actuators, conical actuators exhibit a wide range of bending curvatures and greater flexibility. Experiments demonstrated that by selecting an appropriate cone angle, the suction cups of the conical actuator can grip and move on a wide range of surfaces: flat, non-flat, smooth, or rough. Additionally, by optimizing the suction cup size and pattern of different arm cone angles, the overall grasping performance of the actuator can be significantly enhanced. The success of this experiment provides new design ideas for the creation of the next generation of soft actuators for grasping various objects of diverse shapes.

However, direct-type pneumatic chambers tend to undergo significant expansion deformation under high-pressure gas or fluid drive. To limit the radial deformation of pneumatic soft actuators and improve their driving performance, researchers have conducted a series of explorations. Mosadegh et al. [54] designed a pneumatic soft actuator composed of a series of independent and interconnected chambers. The soft robot is inflated and driven through a “gas network” (a pneumatic network composed of small channels in the elastic material), which allows it to produce complex movements with simple control. However, the movement speed is relatively slow at present, so based on this, the researchers designed a new gas network that reduces the amount of gas required for inflation, thereby improving the driving speed. Gunawardane et al. [55] proposed a new soft pneumatic actuator (SPA) composed of a series of cavities with the same helical angle, capable of simultaneously generating bending and twisting movements. The thin-walled hermetic spiral actuator is directly manufactured using 3D printing without any post-processing. Experiments show that, under the same pressure input, this spiral actuator has a higher mechanical output compared to regular bending actuators. It maintains a certain flexibility and can be used to grasp objects with complex shapes. Kano et al. [56] proposed a new type of sheet-like 2D soft robot called Soft Robot Surface (SRS), which is driven by pneumatic network bending actuators. During testing, the deformation shape of the SRS was reconfigured by controlling the applied pressure. Additionally, the bending angle of both actuators decreased with an increase in the soft surface’s width and thickness. Based on this, they created a soft gripper capable of grasping objects of various sizes, shapes, and stiffness, demonstrating the application of SRS. Wang et al. [57] designed a segmented PneuNets bending actuator-based soft pneumatic glove, which is structured according to the anatomical structure of the human finger. It consists of five segmented PneuNets bending actuators (SPBAs) made of elastomers, each driving the corresponding finger to bend. Tests on the passive bending degree and gripping force of human fingers driven by the glove showed that the design of the soft glove is feasible and has potential for development in hand rehabilitation.

(3) Corrugated Structure Pneumatic Soft Actuators

Corrugated structures generally exhibit higher stiffness in directions parallel to the ridge and valley, while maintaining good flexibility in the axial direction. By utilizing the stiffness characteristics of corrugated structures, pneumatic soft actuators with good stretchability can be designed. Drotman et al. [58] designed a 3D-printable corrugated tube pneumatic actuator. This 3D-printed actuator consists of three chambers connected in parallel, and these chambers rotate around the actuator’s longitudinal axis. This three-chamber 3D-printed actuator can be widely used in soft grippers for handling fragile objects or in the legs of soft quadruped robots. By adjusting the geometry, materials, and pressure, the actuator can be developed to meet the requirements of each application, offering good practical prospects. Kim et al. [59] proposed a soft pneumatic gripper driven by a tendon-driven soft origami pump. The soft finger module is composed of a soft pneumatic actuator, air channel components, and an origami pump. Based on this, the researchers conducted a series of experiments and analyses to evaluate the performance of the actuator, including motion characteristics, frequency response, blocking force, and the relationship between pressure and bending angle. The results indicated that the intrinsic frequency of the pneumatic actuator is about 3 Hz, and the use of tendon-driven principles and pneumatic actuators for the soft origami pump is feasible. Zhou et al. [60] proposed an embodiment with position feedback and force estimation for a pneumatic bellows (PB) actuator, where an internal bellows acts as a position sensor for the PB actuator, and the external bellows functions as the pneumatic actuator. Experiments found that by controlling the input pressure, the pneumatic bellows can generate the desired deformation of the PB actuator, and the internal conductive bellows will experience a change in resistance due to deformation. This provides a feasible solution to the sensing challenges in deformable soft robots with large resistance changes and structural deformations. Yap et al. [61] proposed a new technique for direct 3D printing of pneumatic soft actuators based on fused deposition modeling (FDM) technology. Researchers studied the characteristics of printing materials to simulate the mechanical behavior of the printed actuators. Testing of the actuators’ bending ability, output force, and durability showed that the actuators could lift heavy objects with a high output-to-weight ratio, while also achieving complex movements, suggesting that 3D printed actuators have potential soft robot applications. The corrugated structure pneumatic soft actuators share certain structural similarities with elastic chamber actuators, but their deformation is more concentrated, offering higher driving efficiency and the ability to generate large-scale movements even under small strains in the chambers. Therefore, corrugated structure actuators have great potential for use in soft robotic arms and flexible grippers for large loads.

(4) Folded or Pleated Pneumatic Soft Actuators

Folded or pleated structures are commonly seen in everyday life and exhibit high deformation rates when expanded or folded. By incorporating folded/pleated structures into the design of soft actuators and driving them with a gas medium, large-scale deformation can be achieved. Kim et al. [62] achieved precise sequential deployment and bending motion driven by a single fluid input through a dual origami design. The actuator has good scalability by selectively placing strain-limiting folded layers between small planes of a fluid network. Additionally, the actuator can be automatically manufactured using accessible 3D printing technology. Experiments showed that this dual origami flexible fluidic bending actuator differs from traditional design methods, mainly focusing on motion generation, and offers a wider range of materials for use. This provides broader design ideas for the preparation of small-form-factor soft robots. Li et al. [63] proposed a fluid-driven origami artificial muscle architecture, using various materials and scales for the rapid manufacturing of low-cost artificial muscles. Nylon was used for the linear sawtooth actuators for the skin, and related tests revealed that this linear artificial muscle could generate about 600 kPa of driving stress (approximately six times the sustainable stress of mammalian skeletal muscle). This muscle can be programmed to achieve complex multi-axis movements, offering a new approach to the quick design and low-cost manufacturing of drive systems.

**Figure 3:**
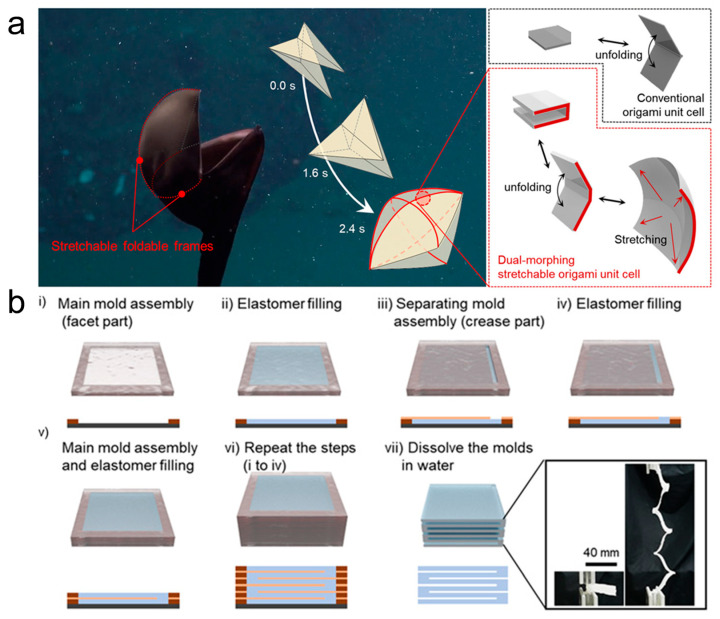
(**a**) Morphing principle of the pelican eel interpreted by the stretchable forms of origami fish base. Red lines indicate the pelican eel’s stretchable and foldable frames. Attached: concept of bioinspired dual-morphing stretchable origami in comparison with conventional origami [66]. (**b**) Fabrication of dual-morphing stretchable origami. Repetitive steps of mold assembly and elastomer filling were conducted to build the architecture of C-channel origami units. A subsequent demolding process was carried out by dissolving the molds in water. The final origami architecture was not only deployable but also stretchable [66].

Actuators enhance a silicone matrix by using materials such as paper or fabric, which have no stretching properties, and achieve movements such as stretching, twisting, bending, and swinging through different folding structure designs. Martinez et al. [64] developed pneumatic soft actuators based on composite materials made of embedded sheets or fiber structures in elastomers. The experiments showed that these actuators have good flexibility, a simple structure, lightweight, and easy to drive, making them widely applicable in practical uses. Li et al. [65] designed a pneumatic soft actuator based on the Miura origami structure. Multiple Miura-folded sheets were connected along their crease lines to form a space-filling structure, with the tubular units in the center filled with a working fluid. Kim et al. [66] were inspired by the dual deformation structure of the pelican eel and created a pneumatic origami actuator with high deformation rates, mimicking the deformational principles of the pelican eel’s stretchable and foldable framework (Figure 3). Feng et al. [67] developed a foldable structure type soft actuator with variable length. Drawing inspiration from the unique movement mode of leeches, they constructed a folding structure for elongation and bending movement patterns. Characterization tests showed that the actuator combines both flexibility and rigidity, making it better suited for addressing issues that traditional constant-length soft grippers struggle with. This actuator has good prospects for real-world applications. Similarly, soft structures can be designed to create high-performance pneumatic soft actuators based on the folding/pleating behavior under negative pressure. Oguntosin et al. [68] created an artificial muscle structure under the soft elastomer actuator (SEA) category, made entirely from soft silicone rubber. In their study, the performance in terms of shortening speed, strain, and pulling force was tested. The maximum contraction strain reached 67%, and the maximum contraction speed was 0.217 s^−1^ under zero load conditions.

Pneumatic soft actuators based on folded/pleated structures have been a hot research topic in recent years, and their high deformation rates provide a wide range of application prospects across various fields. However, folded/pleated structures inevitably lead to stress concentration problems, which may affect the service life of soft actuators. Therefore, it is important to consider stress concentration in the design process of actuators and minimize its negative effects as much as possible.

## 4. Overview of Fabric-Based Pneumatic Soft Actuators

### 4.1. Principles of Fabric-Based Pneumatic Soft Actuators

Fabric-based pneumatic soft actuators are soft-driven devices that use flexible pneumatic chambers made from fabric materials, which achieve mechanical motion through internal pressure changes [69]. Thanks to the flexibility and designability of the fabric structure, they can realize complex programmed flexible movements [70]. These actuators have developed rapidly in the field of soft actuators due to their simple manufacturing process, low cost, high driving force, and fast response time. They have become one of the most widely studied and applied technologies. Although fabric materials are not as high-performance as materials like silicone rubber in terms of tensile modulus, and thus are not suitable for scenarios requiring extremely high driving forces, they show great potential in environments where the demand for driving force is not high, such as in medical rehabilitation gloves and miniature robotic arms [71]. The textile material characteristics of fabric-based pneumatic soft actuators provide unique advantages in terms of deformation and load-bearing capacity, opening new paths for innovative actuator designs. In addition, the influence of fabrics on actuator performance is substantial, primarily due to their distinct physical properties and structural configurations. Fabrics can be categorized into three main types: woven, knitted, and non-woven, each imparting unique characteristics that significantly shape the operational attributes of the actuators constructed from them [72].

Woven fabrics are meticulously crafted through the interlacing of warp and weft yarns, resulting in a highly structured and tightly knit material [73]. This construction endows woven fabrics with remarkable strength and stability, enabling them to effectively distribute stress across their surface. Consequently, actuators fabricated from woven fabrics exhibit enhanced durability and maintain a stable shape and size during the inflation and deflation processes [74]. This stability is crucial for the precise control of actuator movements, ensuring consistent performance. However, the downside of woven fabrics is their relatively low elasticity, which can restrict the range of motion and adaptability of the actuators [75]. Moreover, the dense structure of woven fabrics often leads to poor permeability, impeding the flow rate of gas within the actuator and consequently reducing its response speed.

Knitted fabrics, on the other hand, are characterized by their interlocking loops, which provide them with exceptional elasticity and flexibility [76]. This inherent flexibility allows knitted fabrics to undergo significant deformation in response to external forces, rendering the actuators highly compliant. Such compliance is particularly advantageous in applications where the actuators need to adapt to varying working environments and task requirements [77]. For instance, in rehabilitation devices or wearable robots, knitted fabric actuators can conform closely to the human body, offering a comfortable and natural interaction experience. Despite these benefits, knitted fabrics are generally weaker in terms of strength and have limited durability. Therefore, specific measures must be incorporated into the design and manufacturing processes to enhance the longevity and robustness of these actuators.

Non-woven fabrics are created by bonding fibers together using methods like adhesion, friction, or electrostatic forces [78]. The resultant structure is relatively loose, which bestows non-woven fabrics with excellent air permeability and gas permeability. These properties are highly beneficial for pneumatic soft actuators, as they facilitate rapid gas inflow and outflow, thereby significantly improving the actuator’s response speed [79]. However, non-woven fabrics are typically characterized by their poor strength and elasticity, and their durability is generally inferior to that of woven and knitted fabrics [80]. When utilizing non-woven fabrics for actuator manufacturing, it is imperative to carefully select the type of fabric and optimize the processing techniques based on the specific application scenarios and performance requirements. This approach helps to strike a balance between air permeability and mechanical properties, ensuring that the actuators meet the desired functional criteria.

The selection of fabric type and the consideration of its characteristics play a pivotal role in determining the overall performance of pneumatic soft actuators [81]. During the design and manufacturing stages, it is essential to conduct a comprehensive evaluation of the fabric’s elasticity, permeability, and durability [82]. As research in soft robotics and flexible actuators continues to deepen, the progress in fabric-based pneumatic soft actuators has been significant. Researchers have explored more efficient and precise pneumatic actuation mechanisms by designing soft actuating components with different structures and materials and optimizing pneumatic channels and control systems. A research approach combining computational simulations and experimental verification has been widely used to analyze the working performance and application potential of these devices. These studies have not only advanced the technology of fabric-based pneumatic soft actuators but also provided a solid theoretical foundation and practical guidance for innovative applications in related fields. As these actuators are continuously optimized in design and functionality, they are expected to play an even more critical role in medical assistance, human–computer interaction, wearable technology, and flexible automation.

### 4.2. Research Progress on Fabric-Based Pneumatic Soft Actuators

Fabric-based pneumatic soft actuators have become a research hotspot in the textile and mechanical fields in recent years. Unlike conventional elastomer-based pneumatic soft actuators that are manufactured through complex and time-consuming molding-casting processes or expensive 3D printing, fabric-based pneumatic soft actuators can be easily produced by wrapping a textile shell around a pneumatic chamber or bonding pre-existing composite fabrics with different anisotropic mechanical properties. Additionally, fabric-based pneumatic soft actuators naturally inherit the advantages of textiles, such as lightness, flexibility, compliance, durability, and customization, making them ideal for human–computer interaction and custom wearable applications. However, the efficient design and low-cost manufacturing of fabric-based pneumatic soft actuators and soft textile robots remain long-standing and well-recognized challenges.

**Figure 4:**
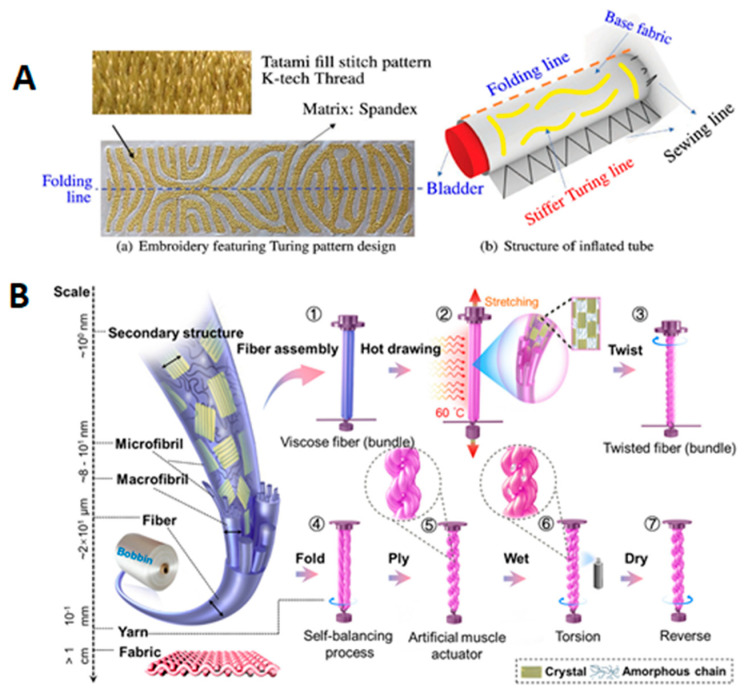
(**A**) a—Embroidery featuring Turing pattern is designed to facilitate bending after pressurization. Tatami fill stitch type is used for this application; b—Structure of inflated tube consists of an embroidered sheet featuring a Turing pattern [84]. (**B**) Design and actuation mechanisms of the viscose/PET yarn actuator [86].

To address this actuator, researchers have made different attempts to break through the limitations of traditional flexible robots in application development. One of the main challenges for textile-based soft robotics technology is the generation of anisotropic mechanics. The anisotropic mechanics of fabric-based pneumatic soft actuators are determined by the differences in the elastic moduli of the fabrics. Therefore, fabric-based pneumatic soft actuators are divided into two parts: a bottom layer and a top layer, sealed in the middle using stitching or heat-sealing processes. The bottom layer material is defined as the strain-limiting layer, which requires a large elastic modulus and minimal strain under load. Typically, woven fabrics are used for the bottom layer due to their structure, which provides minimal mechanical compliance. The top layer material requires a smaller elastic modulus and preferred strain in a certain direction. When internal air pressure is increased in the cavity between the two layers, the difference in elastic moduli causes the bottom fabric to undergo little deformation or no deformation, while the top fabric undergoes significant deformation, causing the actuator to bend toward the side of the bottom layer. In response to this, researchers have designed pneumatic fabric soft robots based on “encoded sewing constraint mechanics.” Guo et al. [83] introduced a unique encoded sewing constraint (ESC) design method for effectively constructing three-dimensional textile shells with programmed global strain constraints for STRs. During seam formation, bonding and strain constraints are achieved simultaneously: different types of stitches represent two different stretch performances, and three monotonic warp-knit fabrics are bonded together using simple and efficient 2D sewing techniques to form 3D constrained textile shells. Based on this, researchers developed a series of multifunctional STRs driven by the ESC method and pneumatics. Experiments show that the unique design of encoded seams with pre-programmed stretch properties not only allows textiles to construct 3D constraint shells using simple 2D manufacturing methods but also greatly simplifies the difficulty in predicting complex 3D deformations. By simply adjusting the stitch characteristics of the seams, high-dimensional programmability of STRs, including shape deformation and expansion sequences, is achieved, which accelerates the application of pneumatic fabric soft robots in safe human–computer interaction, customized wearables, and the prototyping and product iteration of medical rehabilitation devices. Moreover, Tanaka et al. [84] presented a novel computational design and fabrication method for fabric-based soft pneumatic actuators that use Turing patterns, inspired by Alan Turing’s morphogenesis theory. These inflatable structures can adapt their shapes with simple pressure changes and are applicable in areas like soft robotics, airbags, and temporary shelters. It introduces a method to automate this process using advanced numerical optimization to design and manufacture fabric-based inflatable structures with programmable shape-morphing capabilities (Figure 4A).

Secondly, due to the lack of simple, mold-based, easily programmable, and modelable prototyping methods, constructing complex pneumatic channels within fabric-based chambers presents challenges that greatly limit the development of pneumatic actuators made entirely from fabric. Zhang et al. [85] proposed a new design for a gas-driven soft robot using a single-piece fabric, leveraging the fabric’s flexibility and resistance to stretching. They developed a novel pneumatic actuator by embedding air channels within the fabric, achieving high load capacity and high compliance. At the same time, by adjusting the design of the air channels within the fabric, they achieved diverse deformation modes. Experiments showed that pneumatic actuators made from soft yet non-stretching fabric could achieve adjustable working spaces and carry high loads simultaneously. The actuator’s motion modes are programmable, combinable, and capable of fast response with low input pressure. Relevant tests revealed that a robot gripper made from three fabric actuators demonstrated a maximum gripping force of over 150 N and a gripping range exceeding 350 mm, providing a design and analytical foundation for applying non-stretching but soft materials to soft robots to enhance their practicality.

At the same time, researchers have studied different fiber materials, such as functional fibers and smart fibers, to develop actuators with high response speed, high load capacity, and long lifespan. Artificial muscles have received widespread attention for their sensitive responses to environmental changes (such as water or humidity, heat, light, electricity, and magnetism). Fiber-based artificial muscles have been extensively studied in soft robotics, energy engineering, and intelligent systems due to their high specific energy density, simple structure, and flexibility. Peng et al. [86] converted commercial viscose fibers into highly humidity-sensitive artificial yarn muscles with reversible torsional stroke. As shown in Figure 4B, artificial yarn muscles were prepared using a top-down and bottom-up combination, inducing fiber alignment through a heat-stretching process, followed by twisting and plying to form a double-helix yarn muscle structure. This improved the response performance of viscose fiber artificial muscles. Through various textile techniques, yarn muscles were expanded into fabric muscles, realizing complex spatial deformations such as bending, rolling, and twisting. Related tests showed that these yarn artificial muscles had excellent torsional strokes (1752 cm^−1^) and maximum rotational speeds (2100 rpm), comparable to artificial muscles made from carbon-based composites. Heat-stretching-treated viscose fibers exhibited better humidity response performance than original viscose fibers. Fabric muscles, after absorbing water, could achieve rolling.

Similarly, researchers have explored new textile structures inspired by biology to improve the flexibility, stability, and reliability of actuators. Yang [87] et al. proposed an innovative textile structure design inspired by caterpillars. By combining boucle yarn and an innovative three-layer knitted structure, they developed a soft pneumatic actuator based on a layered textile structure with fast response and large bending drive strain. The layered structure design and manufacturing method allow the actuator to have a high deformation capability in the highly elastic region and minimal deformation in the low elastic region, thereby maximizing the conversion of supplied pressure into effective driving strain and work output. Tests showed that the prepared actuator could rapidly generate large bending strain under 50 kPa air pressure, with high power density, good repeatability, and durability. They also proposed a new knitted soft robot inspired by the Venus flytrap [88]. Using knitting technology, they formed different loop structures by varying stitch types and raw materials to integrate sensing performance and specific mechanical properties, proposing a cost-effective digital knitting strategy for rapid programming, manufacturing, and achieving various programmable actuators. Pressure sensors and tensile strain sensors based on knitted structures enable environmental sensing and feedback on robot deformation. Performance tests showed that this soft robot had fast response and good deformation ability, including high driving strain (1400 m^−1^) and high volume power density (212 W·m^−3^).

The recent advancements in fiber materials and textile structures have significantly elevated the performance of soft actuators, as evidenced by the aforementioned studies. These innovations have catalyzed the development of a variety of pneumatic-driven actuators, each characterized by unique operational attributes and application potentials. To elucidate the comparative advantages and limitations of these actuators, the following Table 2 provides a comprehensive analysis of their performance metrics, focusing on critical indicators such as drive force, response speed, and deformation.

## 5. Summary

Soft actuators, as a pivotal technology in the field of soft robotics, encompass interdisciplinary research and development spanning materials science, mechanical engineering, electrical engineering, and computer science. These actuators emulate the motion of biological muscles to achieve material deformation, movement, and mechanical responses, providing a novel alternative to traditional rigid mechanical drives. The design and optimization of soft actuators necessitate a comprehensive consideration of material selection, actuation mechanisms, structural design, and control strategies to ensure efficient operation in confined or specialized environments.

With regard to structural design, soft actuators are continually refined to meet diverse application requirements. For example, fiber-reinforced pneumatic soft actuators enhance mechanical performance and range of motion by integrating fibers or fabrics into their chamber structures. Wave-like and folded/pleated pneumatic soft actuators achieve large deformations and movements through specialized geometric configurations. In terms of control strategies, the precision and response speed of soft actuators are critical performance metrics. Researchers are investigating advanced sensors, control algorithms, and machine learning techniques to improve actuator performance. Furthermore, the integration of feedback control systems enables soft actuators to monitor and adjust their states in real time, ensuring optimal performance under varying operating conditions.

The combination of fabric-based pneumatic soft actuators with flexible sensors represents a highly promising approach, enabling the creation of highly responsive and multifunctional systems. Pneumatic actuators exhibit excellent adaptability and compliance, while flexible sensors provide real-time feedback on strain, pressure, and deformation. By embedding sensors directly into fabric structures, seamless integration of actuation and sensing can be achieved, allowing for more precise motion control and expanded functional capabilities. For instance, this synergy could lead to the development of soft robots capable of sensitive environmental interactions, such as adaptive grippers or wearable robots that respond intuitively to body movements. Moreover, this integration facilitates the creation of intelligent systems that dynamically adapt to external forces. In healthcare, real-time monitoring of muscle or joint activity could enhance rehabilitation devices; in the automotive industry, it could advance prosthetic or exoskeleton technologies. Thus, combining fabric-based pneumatic actuators with flexible sensors not only extends the functional capabilities of these systems but also paves the way for smarter and more adaptive devices, revolutionizing robotics and human–machine interaction.

Nevertheless, current flexible pneumatic actuators still encounter numerous unresolved challenges. First, system reliability remains inadequate, particularly concerning fatigue, durability, and damage resistance. Due to their operation under repeated large deformations, they are prone to fatigue damage and performance degradation. Additionally, the puncture resistance of soft materials is often insufficient, and once damaged, their actuation performance may significantly decline or fail entirely. Second, the response speed and deformation range of flexible pneumatic actuators require improvement. Some actuators exhibit limited deformation and slow response/recovery speeds, rendering them unsuitable for complex application scenarios. Third, their highly nonlinear behavior and hysteresis effects render traditional finite element models and control algorithms insufficient for capturing their complex microstructures and mechanical responses, increasing the difficulty of simulation studies. For instance, the multiscale properties of fabric-based materials, fiber-yarn contact friction, and rearrangement during deformation are challenging to accurately describe using conventional finite element models. Finally, pneumatic supply remains a major constraint for widespread adoption. Most flexible pneumatic actuators depend on external air sources, and the required pressure is still relatively high, limiting their mobility and flexibility. Although various mobile pneumatic supply solutions have been proposed, mature technologies still fall short of fully resolving these issues.

Future research will continue to address the challenges of multi-directional expansion and large deformation, the contradiction between inherent flexibility and driving force, as well as insufficient control accuracy and response speed. With the development of new materials, innovative structural designs, improved driving technologies, and advanced control algorithms, the development of soft actuators and flexible robots will become more diverse and intelligent, bringing new innovations and application possibilities to various fields. Additionally, with the progress of manufacturing technologies such as 3D printing and flexible electronics, the manufacturing of soft actuators will become more efficient and cost-effective, further promoting their wide application in areas such as healthcare, rescue operations, exploration, and human–machine interaction. Additionally, with the progress of manufacturing technologies such as 3D printing and flexible electronics, the manufacturing of soft actuators will become more efficient and cost-effective, further promoting their wide application in areas such as healthcare, rescue operations, exploration, and human–machine interaction.

## Figures and Tables

**Table 1:** Performance and application comparison table of soft actuators with different driving modes.

Actuator Type	Advantages	Disadvantages	Applications
**Liquid-driven Actuators**	- Significant force generation - High energy density - Suitable for applications requiring high force output (e.g., flexible robotic arms, bionic devices)	- Requires complex fluid systems - Slower response times - Sensitive to environmental humidity	- Flexible robotic arms - Bionic devices - High-force applications
**Temperature-driven Actuators**	- Simple structure - Fast response speed - No external power source needed - Environmentally friendly and pollution-free	- Performance highly dependent on ambient temperature and material thermal conductivity - Limited precision and reliability in variable environments	- Micro-mechanical systems - Biomedical devices - Autonomous or environment-powered soft robotics
**Magnetic-driven Actuators**	- Precise control - Fast response speed - Remote operation capabilities - Tubing-free operation	- Requires high-strength magnetic field sources - Potential interference with other devices - High cost for large-scale systems	- Applications requiring remote control - Micro-robots - Medical instruments
**Electric-field-driven Actuators**	- High energy efficiency - High control precision - Fine control with rapid response - Easy sensor integration	- High operating voltages (kV-scale) - Requires complex electrical systems - Sensitive to environmental factors (humidity, temperature)	- Applications requiring fine control and rapid response - Micro-robots - Flexible sensors
**Pneumatic-driven Actuators**	- Intrinsic safety - Strong environmental adaptability - Simple and lightweight design	- Requires external compressor systems - Slow response - Nonlinear control complexity - Potential noise issues - Gas leakage susceptibility	- Soft grippers - Wearable devices - Rehabilitation robotics

**Table 2:** Performance comparison of different types of pneumatic soft actuator.

	Conventional Pneumatic Cylinders	Pneumatic Muscle	Pneumatic Thin-film Actuators	High-Speed Pneumatic Valve Actuator	Fabric-Based Pneumatic Soft Actuator
Drive Force (N)	500–5000	100–2000	1–50	100–1000	2–100
Stroke/Deformation	10–1000 mm	20–300% contraction	1–10 mm	1–5 mm	100–400% elongation
Response Speed (ms)	50–200	100–500	10–50	1–10	80–400
Typical application scenarios	Industrial automation	Robots, rehabilitation equipment	Microfluidics, sensors	Precision control, injection system	Wearable haptic suits, Smart textiles for healthcare, Adaptive ergonomic supports
Reference	[89]	[90]	[91]	[92]	[93]

## Data Availability

No new data were created or analyzed in this study. Data sharing is not applicable to this article.
